# Supplementary figures and images for: Sex Chromosome Evolution, Heterochiasmy, and Physiological QTL in the Salmonid Brook Charr Salvelinus fontinalis
Source: G3 (Bethesda). 2017 Jun 16;7(8):2749–62. doi: 10.1534/g3.117.040915 (PMC5555479; doi:10.1534/g3.117.040915)

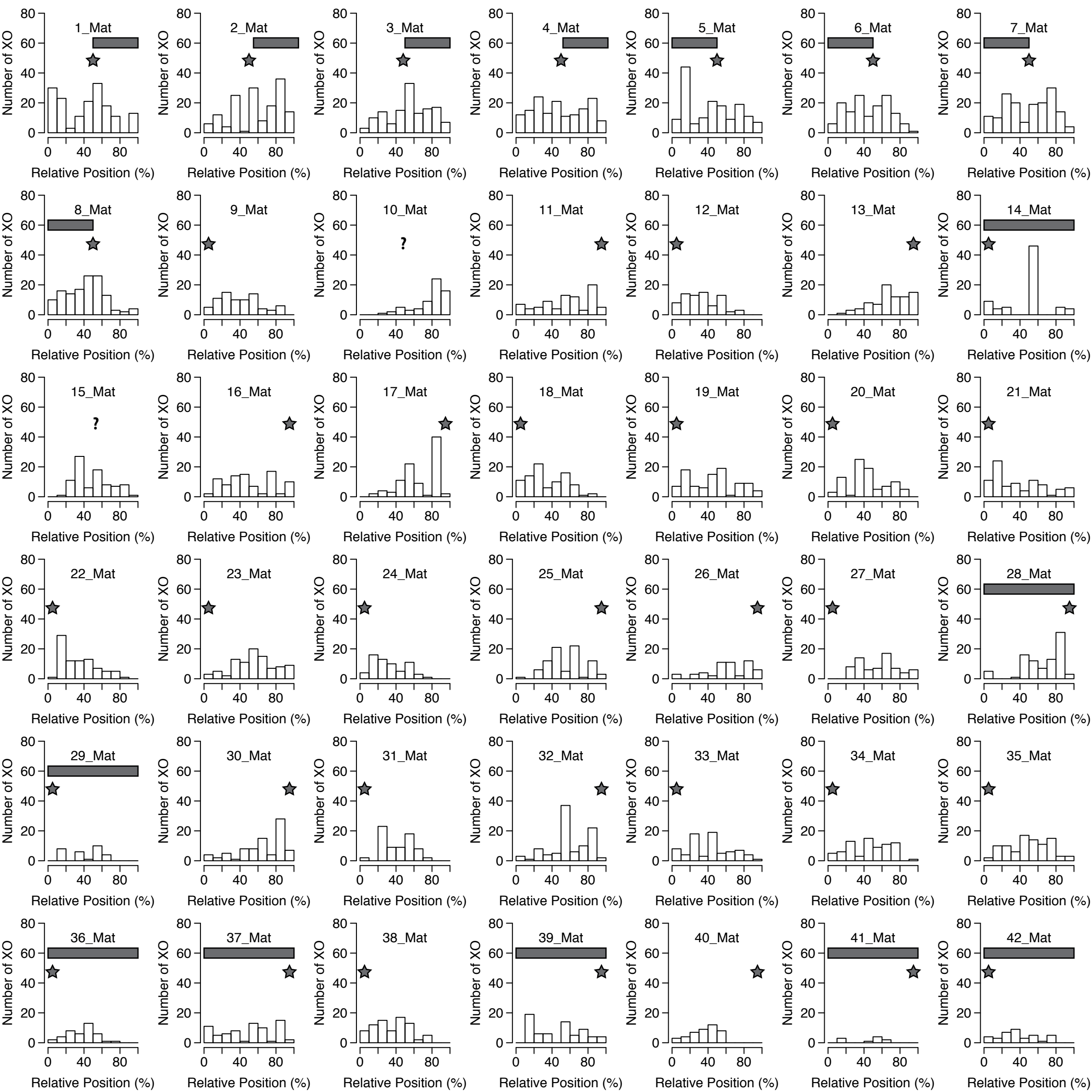

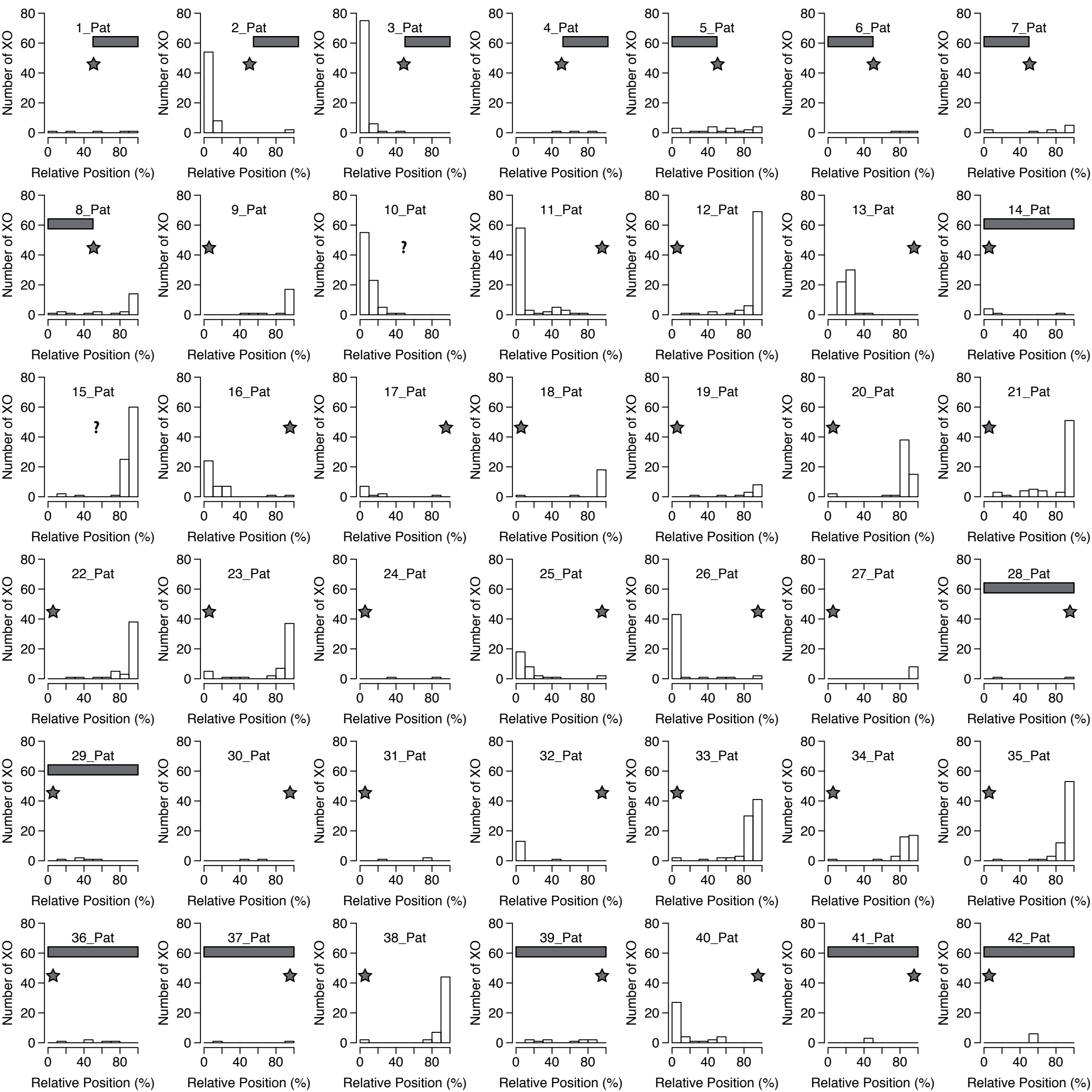

Supplement: Supplementary file 1 [file 2749FigureS1.pdf]

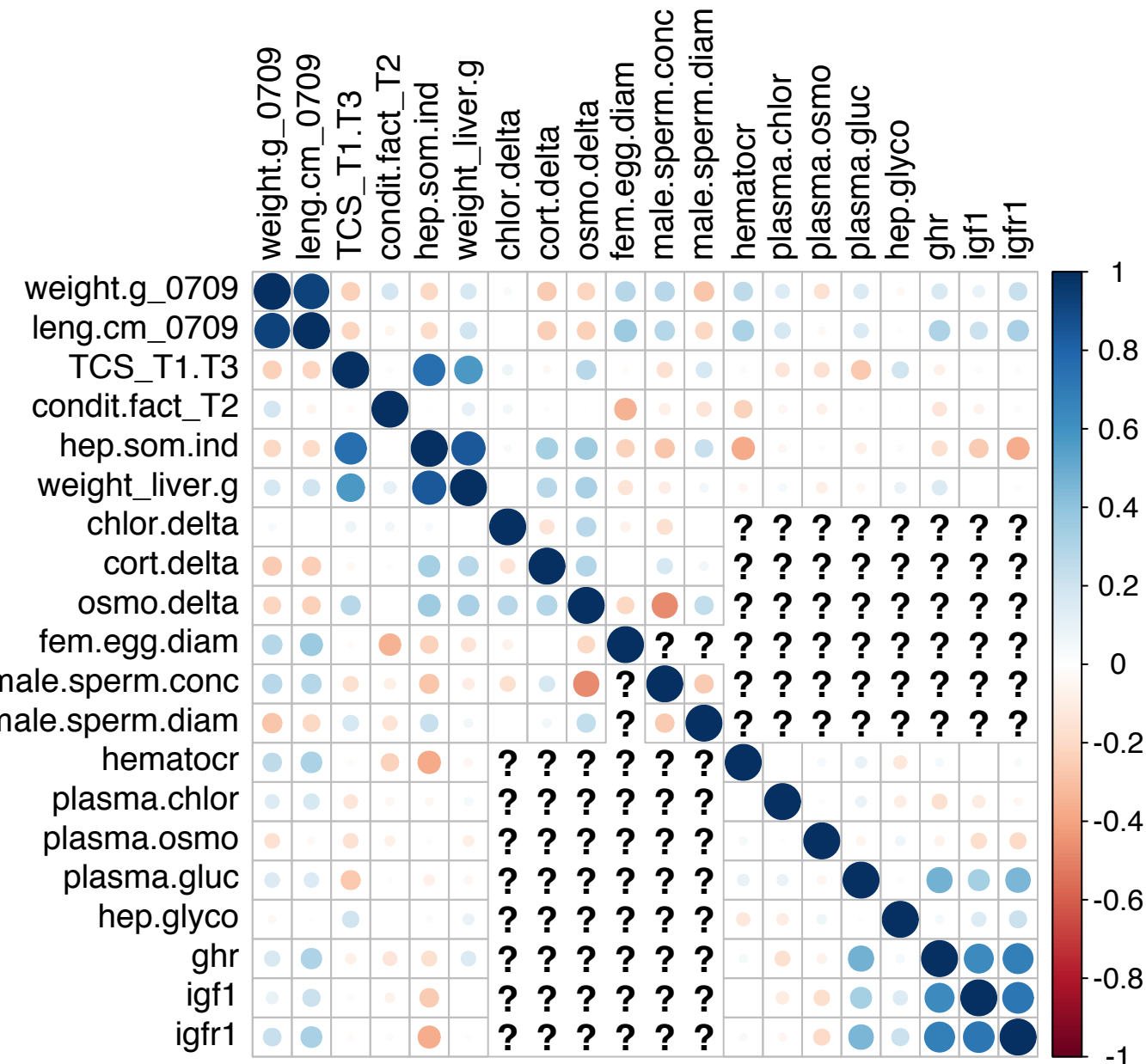

Supplement: Supplementary file 2 [file 2749FigureS2.pdf]
