# Supplementary material for: Sex Chromosome Evolution, Heterochiasmy, and Physiological QTL in the Salmonid Brook Charr Salvelinus fontinalis
Source: G3 (Bethesda). 2017 Jun 16;7(8):2749–62. doi: 10.1534/g3.117.040915 (PMC5555479; doi:10.1534/g3.117.040915)
Supplement: Supplementary file 3 [file 2749TableS1.pdf]

Phenotype	Female Avg	Female Stdev	Female (n)	Male Avg	Male Stdev	Male (n)	Sex as a covariate
Weight (g) at T1	65.1	16.3	78	80.7	21.4	91	yes
Weight (g) at T2	131.7	26.4	78	160.4	35.7	91	yes
Weight (g) at T3	230.9	57.8	78	272.7	60.7	91	yes
SGR (T1-T2)	1.2	0.1	78	1.2	0.1	89	no
SGR (T2-T3)	0.6	0.2	78	0.6	0.2	89	no
SGR (T1-T3)	0.8	0.2	78	0.8	0.2	91	no
Length (cm) at T1	18.5	1.5	78	19.8	1.6	91	yes
Length (cm) at T2	22.2	1.5	78	23.8	1.6	89	yes
Length (cm) at T3	27.2	2.1	78	28.7	1.8	91	yes
Condition factor at T1	1.0	0.1	78	1.0	0.1	91	no
Condition factor at T2	1.2	0.1	78	1.2	0.1	87	no
Condition factor at T3	1.1	0.1	78	1.1	0.1	91	no
Liver weight (g)	3.7	1.9	78	3.3	1.7	91	yes
Hepatosomatic index	1.7	0.8	78	1.3	0.7	91	yes
Hematocrit (% red blood cells)	35.4	3.6	32	38.1	4.2	42	yes
Change in cortisol (µg/dL plasma)	8.8	5.5	45	0.5	2.7	47	yes
Change in osmolality (mmol/kg)	9.0	17.2	44	-4.6	13.0	47	yes
Change in chloride (mmol/L)	-1.7	7.1	45	-0.8	3.9	47	no
Female egg diameter (mm)	4.0	0.2	42	NA	NA	NA	sex specific
Male sperm concentration	NA	NA	NA	63813	17301	46	sex specific
Male sperm diameter (µm)	NA	NA	NA	2.9	0.0	46	sex specific
Plasma chloride (mmol/L)	134.4	6.3	30	133.4	4.8	41	yes
Plasma osmolality (mmol/kg)	322.9	9.2	30	319.6	11.0	41	no
Plasma glucose (mg/mL plasma)	0.7	0.2	30	0.7	0.1	41	no
Hepatic glycogen (mg/g liver)	104.6	13.2	30	113.4	14.7	41	yes
ghr	-4.6	1.7	30	-3.7	2.1	39	no
igf1	-5.5	1.5	30	-4.5	2.2	39	yes
igfr1	-5.6	1.4	30	-4.4	1.7	39	yes

Note: T1-T3 = time points 1-3 (2009/05, 2009/07, 2009/11); SGR = specific growth rate; *ghr, igf1* and *igfr1* are all log2 normalized quantities.
